# The Effect of Rhythmic Breathing on the Severity of Sternotomy Pain after Coronary Artery Bypass Graft Surgery: A Randomized Controlled Clinical Trial

**DOI:** 10.1155/2021/9933876

**Published:** 2021-06-10

**Authors:** Hassan Babamohamadi, Masoumeh Karkeabadi, Abbasali Ebrahimian

**Affiliations:** ^1^Nursing Care Research Center, Semnan University of Medical Sciences, Semnan 3513138111, Iran; ^2^Department of Nursing, Faculty of Nursing and Midwifery, Semnan University of Medical Sciences, Semnan 3513138111, Iran; ^3^Student Research Committee, Semnan University of Medical Sciences, Semnan 3513138111, Iran; ^4^Emergency Medicine Group, School of Medicine, Qom University of Medical Sciences, Qom, Iran

## Abstract

**Background:**

Moderate-to-severe pain is reported in up to 75% of the patients in the first 48 hours after cardiac surgery. Evidence suggests that distraction is an effective nursing intervention for controlling short-term and transient pain. Distraction can be achieved by various techniques, including progressive muscle relaxation, meditation, and rhythmic breathing (RB). The present research aimed at evaluating the impacts of RB on the severity of sternotomy pain after Coronary Artery Bypass Graft (CABG).

**Methods:**

This randomized, controlled clinical trial was conducted on 60 patients after CABG surgery at the open-heart surgery Intensive Care Unit (ICU) of Kowsar Hospital, affiliated to Semnan University of Medical Sciences in Semnan, Iran. The patients were selected through convenience sampling and randomly assigned to two groups, including (1) intervention or RB and (2) control groups. RB was performed in the intervention group every 12 hours (9 a.m. and 9 p.m.) for three consecutive days after the surgery. The control group received only routine care for pain control (opioid analgesics) with no additional interventions. The severity of pain was measured every day in both groups of patients before and after the interventions using the Visual Analog Scale (VAS).

**Results:**

The mean postintervention pain scores were significantly different from the mean preintervention scores in the intervention group (*p* < 0.05). The changes in the mean pain score in the intervention group were also significantly different from the corresponding changes in the controls (*p* < 0.05).

**Conclusion:**

Based on the results, the severity of pain after the intervention was significantly lower in the RB group compared to the control. RB was found to be an effective technique for reducing the patients' pain and is therefore recommended as a post-CABG pain control technique. Iranian Registry of Clinical Trials: this trial is clinically registered with IRCT20120109008665N7, registered 3 September 2018.

## 1. Introduction

Open-heart surgery is currently performed in many countries to increase the survival and quality of life of patients with cardiac diseases [1]. Coronary Artery Bypass Graft (CABG) is the most common open-heart surgery in the US with 156,931 cases in 2016, which shows an increase of 6.1% compared to the preceding four years, and 63% of them were nonelective [2]. In Iran, over 30,000 open-heart surgeries are performed in different medical centers every year [3], of which 60% are CABG [4]. Despite being a successful cardiac treatment, heart surgery is still a traumatic event that can lead to a wide range of complications, including severe pain in the surgery site, stroke, pulmonary edema, pericarditis, and postsurgery depression [5–9].

Several factors contribute to the pain following open-heart surgery, including sternotomy, harvesting saphenous vein, harvesting internal thoracic artery, and inserting various drains [10]. The patients' pain can prevent their effective coughing, deep breathing, and cooperation in physiotherapy; cause the retention of pulmonary secretions; and lead to complications such as pulmonary atelectasis, pneumonia, and respiratory failure [11, 12]. By stimulating the sympathetic nervous system and raising epinephrine and norepinephrine levels, pain can also increase cardiac workload, create an imbalance in oxygen supply and demand, and consequently lead to ischemia and myocardial infarction [13]. Improper pain control after cardiac surgery can cause a high incidence of Poststernotomy Pain Syndrome (PSPS) [14]. PSPS is hard to treat and affects the quality of life of the patients and their relatives in the long term [15–17].

According to medical personnel, patients are not in great pain after sternotomy because the tissue damage that occurred is negligible and the patients are minimally mobile after surgery. Nevertheless, Lahtinen reported moderate-to-severe pain (pain score of 4 to 10) in up to 75% of the patients in the first 48 hours after cardiac surgery [18]. Thus, effective pain management will result in faster recovery, reduced postsurgery complications and length of stay, and increased patient satisfaction.

A variety of medicinal and nonmedicinal therapies have been proposed for pain management. Medicinal methods such as opioids are currently used to alleviate pain in cardiac patients but are not desirable as the first line of treatment because of their costs and adverse effects on different body parts, which increase patient mortality and morbidity [19]. Hence, nonmedicinal pain control interventions are preferred because they reduce the need for medications [20]. As such, various nursing measures have been used as Complementary Therapy Methods (CTM) to help patients [21, 22].

Studies indicate that distraction is an effective nursing intervention for controlling short-term and transient pain by increasing endorphins [23, 24]. Quoting several studies, Malloy and Milling reported distraction as one of the oldest psychological interventions for pain relief with remarkable effects [25]. By balancing the anterior and posterior hypothalamus and reducing the activity of the sympathetic nervous system and the secretion of catecholamine, distraction can mitigate stress-induced muscle tension and physiological side-effects such as hypotension, heart rate, and muscular spasms [26]. Another advantage of distraction in clinical settings is that patients can perform this technique independently. Thus, in combination with analgesics, it provides comprehensive pain relief. Distraction can be achieved by various techniques, including progressive muscle relaxation, mandibular relaxation, meditation, and rhythmic breathing (RB) [27].

RB is one of the distraction methods that make patients voluntarily distract themselves from a painful stimulus and thus help control their pain [28, 29]. Distraction is based on the idea that, with diverse and sufficient sensory stimuli, the reticular formation in the brainstem can choose to inhibit or ignore the transmission of such feelings as pain [30]. In this technique, the perception of pain is minimized through distraction-induced reduced alertness [31]. Moreover, RB mitigates pain, anxiety, and stress by increasing the activity of the parasympathetic nervous system via the vagus nerve and increasing the inhibitory function of the Gamma-Aminobutyric Acid (GABA) receptors in the brain pathways that are vital to perceiving fear, emotional regulation, and stress response. The advantages of using this technique include its simplicity, low costs, noninvasiveness, safety, and long-term application [28, 32].

Several studies have reported the effect of RB on reducing pain [28, 33–37]. For example, Farzin Ara et al. showed that RB reduced pain from 7.2 ± 3.7 to 5.9 ± 1.1 following orthopedic surgeries [33]. Borzou et al. showed that RB can help reduce pain and the frequency of analgesic administration in patients after orthopedic surgeries [34]. Furthermore, Borzou et al. showed that RB is an effective method for relieving pain due to hemodialysis vascular needles [28]. Lalegani et al. (2013), Bozorg-Nejad et al., and Park et al. showed that RB can significantly reduce the severity of pain during the dressing of burns [35–37].

Sternotomy site pain is the patients' most common complaint and probably the most severe postoperative pain experienced, which can be associated with increased patient morbidity and mortality [38]. Patients undergoing open-heart surgery experience the worst possible pain during coughing and deep breathing, and their pain is inadequately controlled and prevents them from deep breathing and effective coughing despite medicinal interventions such as the administration of nonsteroidal anti-inflammatory medications and opioids [39]. Given the lack of research on the effect of this technique on patients' pain following CABG, the present study was designed and conducted to determine the effect of RB on post-CABG sternotomy pain control.

## 2. Methods

### 2.1. Study Design and Participants

**Figure 1:**
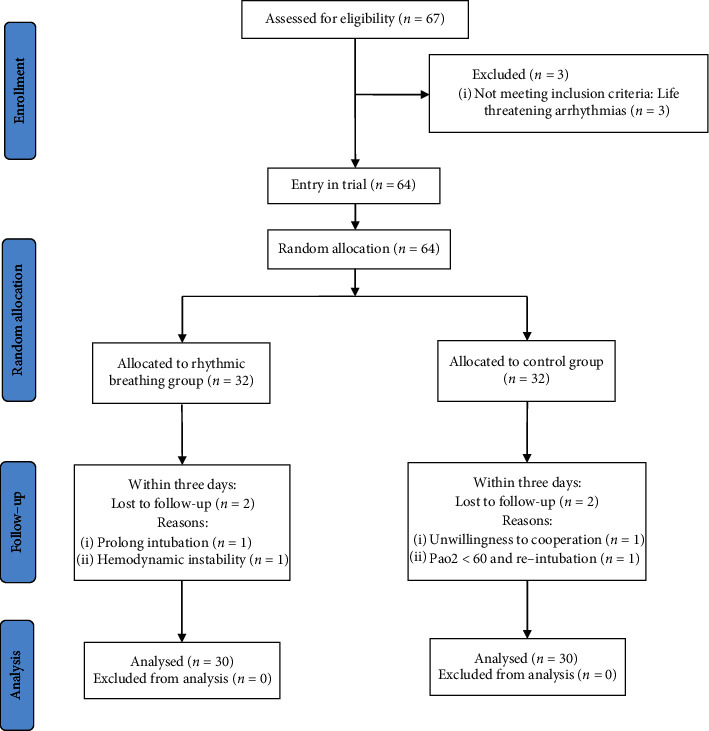
CONSORT flowchart of the study.

This parallel randomized, controlled nonmasked clinical trial was conducted between September 2018 and September 2019 on patients undergoing CABG surgery at Kowsar Hospital, affiliated to Semnan University of Medical Sciences in Iran. Following a preliminary study with ten patients from each group (RB and control), the mean and standard deviation of the severity of pain were found as 1.3 ± 0.65 in the RB group and 2 ± 0.8 in the control group. Then, taking into account 95% confidence interval and 80% test power and using equation (*n*=((*z*_*1-α/2*_ *+* *z*_*1-β*_)^*2*^ *×* *(δ*_*1*_^*2*^ *+* *δ*_*2*_^*2*^*))/(μ*_*1*_ *-* *μ*_*2*_)^*2*^), the sample size was determined as 30 patients per group. Using *G*∗power, the effect size was estimated as 0.96 (means: difference between two independent means). A total of 67 patients entered the study, of whom seven withdrew for various reasons, and the data from 60 patients (30 per group) were ultimately analyzed. The patients who fulfilled the inclusion criteria were included in the study and were then randomly assigned to the groups using random blocks of A for the experimental group (RB) and B for the control group. Since patients who practice RB are easily distinguished and the researcher taught the patients how to breathe rhythmically, it was not possible to perform blinding.  Figure 1 presents the flow chart of the participants.

The study inclusion criteria were as follows: undergoing elective CABG surgery, age 35–80 years, stable hemodynamic status (blood pressure>90 mmHg, and 50 < pulse rate < 110), no previous history of CABG, no life-threatening arrhythmias, and no use of Cardiopulmonary Bypass (CPB). The exclusion criteria were as follows: having a history of diabetes, prolonged intubation (more than 24 hours after surgery), dependence on inotropic agents for hemodynamic stability after extubation, PaO_2_ < 60 mmHg without receiving oxygen via the nasal cannula or mask, renal dysfunction before and after surgery, history of chronic pain, and the need for other pain control methods such as music or massage therapy.

### 2.2. Ethical Considerations

In terms of ethical considerations, the Ethics Committee of Semnan University of Medical Sciences approved the present research (IR.SEMUMS.REC.1397.138). This study was also registered in the Iranian Registry of Clinical Trials (IRCT20120109008665N7). After introducing himself to the patients, the researcher briefed the participants on the research objectives and procedures, assured them of the confidentiality of their information and their right to withdraw from the study at their own discretion and responded to their questions. The subjects then signed written informed consent forms for participation.

### 2.3. Interventions

RB technique was performed for the intervention group. RB was individually taught to the intervention group before surgery until they could perform it independently and correctly. The day after surgery, upon gaining full consciousness and hemodynamic stability, the patients were asked to close their eyes in the supine position, inhale through the nose, then hold their breath, and exhale through the mouth, while counting from 1 to 3 in each step. All the patients in the intervention group were trained to focus only on air entry and exit while breathing, and they were asked to perform RB once every five minutes, lasting one minute each time, for 20 minutes (totally four times every 20 minutes) [32]. The patients received supplemental oxygen during the intervention if required. Supervised by the researcher, RB was performed every 12 hours (9 a.m. and 9 p.m.) for three consecutive days. The control group received only routine care (opioids, such as morphine) with no additional interventions. The routine care given to both groups was the same.

### 2.4. Data Collection

Data were collected using a two-part questionnaire. The first part dealt with the patients' demographic details, including age, gender, education, body mass index (BMI), underlying diseases, duration of hospitalization before and after surgery, history of smoking and opioid use, amount of opioid injection, on-pump duration, ejection fraction, duration of mechanical ventilation, and the number of grafts and chest tubes. Surgery was performed using a cardiopulmonary pump in both groups. Also, the method of anesthesia, anesthesia medications, and fluid therapy during and after surgery were the same for all the patients. Any changes in routine instructions were recorded by the researcher.

In the second part, the patients' pain score data were collected by the Visual Analog Scale (VAS). Pain was measured at 9 a.m. and 9 p.m. before and after RB performance in both groups for three days. The patients were asked to express their pain severity by choosing a number between 0 (no pain) and 10 (most severe pain). VAS categorizes the severity of pain into four groups: 0 = no pain, 1–3 = mild pain, 4–7 = moderate pain, and 8–10 = severe pain. In one study, Alghadir et al. confirmed the reliability of VAS with an intraclass correlation coefficient of 0.97 [40]. In the present study, the reliability of VAS was confirmed in a pilot study on ten patients with Cronbach's alpha of 0.94.

### 2.5. Statistical Analysis

Data were analyzed with a per-protocol approach in SPSS-19 (SPSS Inc., Chicago, IL, USA) at a significance level of 0.05. The study data were described, grouped, and compared in absolute and relative frequency tables. The results of the Kolmogorov–Smirnov test confirmed the normal distribution of the data. The independent sample *t*-test was used to compare the two groups in terms of age, BMI, smoking, on-pump duration, ejection fraction, mechanical ventilation, hospitalization before and after surgery, mean severity of pain before and after intervention, difference between the mean values preintervention and postintervention in each group, and the amount of morphine used. The Chi-square test was used to compare the absolute and relative frequencies in terms of the patients' gender, education, drug use, underlying diseases, number of grafts and chest tubes, and pain intensity between the two groups. The repeated-measures analysis of variance was used to assess the effect of time and the interaction effect of group and time on the mean severity of pain three days after surgery.

For analysis of variance with repeated measures, the following statistical presuppositions were examined first: quantitativeness of the dependent variable, elimination of outliers, normal distribution of the dependent variable distribution, and confirmation of sphericity of the groups with Mauchly's statistics, and three factors of intervention (versus control), time point during the day (difference between before and after scores at 9 a.m. and 9 p.m.), and days (first, second, and third) were entered into the analysis.

## 3. Results

### 3.1. Participants' Characteristics

The present study recruited 60 patients after CABG surgery. The patients' mean age was 61.58 ± 9.7 years, and the majority (80%) were male.  Table 1 presents the demographic and surgery details of the patients in both groups. No significant difference was found between the two groups in terms of demographic and surgery details (*p* > 0.05). One-third of the patients in the intervention group and more than half in the control group had several underlying diseases, and hypertension was the single most frequent underlying disease in them.

### 3.2. Severity of Pain

**Figure 2:**
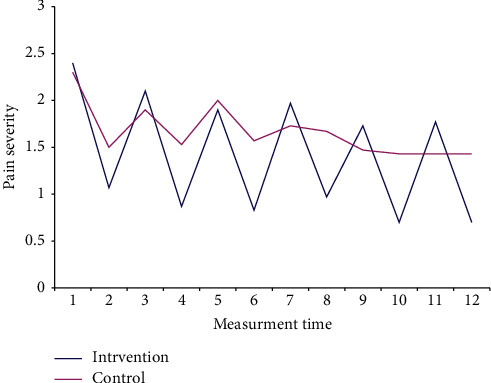
Mean scores of the pain severity after CABG.

The independent *t*-test results showed no significant difference between the two groups in the severity of pain in the first three days after surgery before beginning the intervention, except on the third night (*p* > 0.05). Meanwhile, the severity of pain after the intervention, as measured on both occasions (9 a.m. and 9 p.m.) in all three days, was significantly lower in the RB group compared to the controls (*p* < 0.05) (Table 2, Figure 2). The results of the Chi-square test revealed significant differences between the two groups in terms of the severity of pain (no pain, mild pain, and moderate pain) before and after the intervention (*p* < 0.05). None of the patients in both groups had severe pain (Table 3). There were significant differences between the two groups in terms of the mean difference in the severity of pain before and after the intervention (*p* < 0.05) (Table 4). The results of the repeated-measures ANOVA showed a significant difference between the two groups in the mean difference in severity of pain after the intervention, such that the severity of pain was lower in the RB group compared to the controls. In other words, the intervention reduced the severity of pain significantly over time (*F*(1,58) = 137.3, *p* < 0.001) (Table 5). The mean and standard deviation of the dose of morphine administered was 3.40 ± 1.97 mg in the RB group and 5.30 ± 2.57 mg in the control group. The *t*-test results showed no significant difference between the two groups in the dose of morphine administered (*p* = 0.604).

## 4. Discussion

The present findings on the effect of RB on post-CABG sternotomy pain control confirmed that RB was effective in reducing the severity of sternotomy pain after surgery in the intervention group. To the best of the authors' knowledge, this is the first study to investigate the effect of RB on post-CABG sternotomy pain.

After the intervention, the severity of pain, as measured on both occasions (9 a.m. and 9 p.m.) in three consecutive days following the surgery, was significantly lower in the intervention group compared to the controls. The results of the repeated-measures ANOVA showed a significant difference between the two groups in the mean difference in the severity of pain over the three days, which was lower in the intervention group. In agreement with this result, several studies conducted on the effect of RB on pain have also shown that RB can help reduce pain and the number of analgesics administered after surgery [28, 33, 34]. The results of a study conducted in Iran by Farzin Ara et al. (2018) to compare the effect of reciting the word “*Allah*” and performing RB on postoperative pain in orthopedic patients showed that, compared to the control group, the group reciting *Allah*, followed by the group performing RB, had lower mean severities of pain. They concluded that both methods can be used to reduce pain after orthopedic surgery [33]. The results obtained by Borzou et al. in Malayer, Iran, also showed the effectiveness of RB on the severity of pain after orthopedic surgery and the dose of analgesics administered [34]. The results obtained by Borzou et al. in Hamadan, Iran, showed the effect of RB in reducing the severity of pain caused by hemodialysis vascular needles, as well [28]. Lalegani et al., Bozorg-Nejad et al., and Park et al. confirmed the positive effect of RB on pain reduction during redressing of burns [35–37]. The results reported by Marsdin et al. in their study titled “The Effect of Audio and Video Distractions on Reducing Lithotripsy Pain” showed that there was a significant difference in the perception of pain and distress between the distraction and control groups [41]. The results obtained by Esmaeili et al. and Valizadeh et al. on the effect of regular breathing exercise and music on the pain of inserting intravenous (IV) lines during blood infusion showed that both these methods significantly reduced children's pain, although music was more effective than breathing exercise [42, 43]. Bageriyan et al. compared the effects of bubbling and regular breathing exercise on reducing venipuncture pain in school children admitted to the thalassemia center of Kerman, Iran. Their results showed no significant difference between the two groups in the mean score of pain [44]. The results of a study conducted by Vakilian and Keramat to compare the effects of aromatherapy with lavender and breathing techniques on reducing labor pain showed that the mean change in the severity of pain before and after the intervention was significantly different in the breathing technique group [45].

Nevertheless, the results obtained by Slade showed that these techniques are less effective than expected in reducing pain [46]. According to Mehdizadeh, most studies have highlighted the beneficial and positive effects of breathing and neuromuscular techniques, but there are reports indicating their ineffectiveness [47], including one by Pugh et al., which argued that breathing techniques exhaust the mother and delay childbirth [48].

The results showed no significant difference between the two groups in the dose of morphine administered (1.97 mg in the RB group and 2.57 mg in the control group), but this difference was clinically significant. Other studies reported a significant difference between their intervention and control groups in the number of analgesics received after surgery [33, 34], which disagrees with the present findings. This disagreement can be attributed to the different target populations, patients' gender, and type of intervention or surgery.

In the present study, the severity of pain was significantly higher in the RB group than the control group at 9 p.m., three days before the intervention, but after the intervention, a significant reduction was observed in the severity of pain in the RB group compared to the controls. This important finding is indicative of the effect of RB on reducing pain, which was also confirmed by the repeated-measures ANOVA results.

The results of pain intensity in patients in both groups also revealed that the pain intensity in the RB group significantly decreased after the intervention compared to that in the control group at 9 a.m. and 9 p.m. for three consecutive days. According to the results, although the effect of time on reducing patients' pain intensity should not be ignored, the intervention (RB) significantly affected patients' pain intensity.

According to the researchers, it is necessary to consider factors affecting the perception of the severity of pain. The nature and severity of postoperative pain depend on the size and amount of incision and type of surgery. In addition, the perception of pain depends on ethnicity, culture, beliefs, personal experience of pain, and personality [49]. The factors affecting pain in patients undergoing CABG include the duration of CPB, gender, age (less than 60 years), duration of surgery (more than two hours), and surgery site (thoracic surgery) [16]. Not much evidence supports that the duration of CPB<60 minutes reduces pain. Nonetheless, the release of various cytokines (known as proinflammatory mediators) caused by CPB contributes to pain [50]. Further studies are recommended to further investigate these items.

### 4.1. Study Limitations

The main limitation of the present research included the reluctance of the patients to follow the instructions provided for performing the breathing exercises owing to their improper psychological status. Some measures were thus taken to encourage them to cooperate. A natural limitation of the present study was also associated with the subjective nature of pain, which caused differences in the degree of pain reported by different individuals because pain severity is a patient-reported outcome. The unicenter type of this study and its small sample constituted other limitations, which restricts the external validity of the findings and prohibits their generalizability to other centers. It is recommended that further research be performed with larger samples and more prolonged follow-ups to obtain more accurate results on the effects of rhythmic breathing on pain after CABG with sternotomy.

## 5. Conclusions

The results confirmed that RB is effective in reducing the severity of sternotomy pain after CABG surgery. Given the importance of the management of pain as the fifth vital sign and to prevent the side-effects and problems caused by the lack of proper pain control, especially in patients after CABG, RB can be recommended as a simple, safe, and inexpensive method in the form of an independent nursing activity in conjunction with other medical measures for reducing the severity of pain in patients after CABG surgery.

## Figures and Tables

**Table 1:** The demographic and surgical-related characteristics of the participants in the rhythmic breathing/control groups.

Groups characteristics	RB (*n* = 30) *N* (%)	Control (*n* = 30) *N* (%)	*p* value ^*∗*^
*Gender*			
Male	24 (80)	27 (90)	*X* ^2^ = 1.17, 1, *p* = 0.278
Female	6 (20)	3 (10)	

*Level of education*			
Below high school diploma	28 (93.3)	28 (93.3)	*X* ^2^ = 0.000, 1, *p* = 1
Higher education	2 (6.7)	2 (6.7)	

*Opium consumption*			
Yes	9 (30)	15 (50)	*X* ^2^ = 2.50, 1, *p* = 0.114
No	21 (70)	15 (50)	

*Underlying disease* ^*a*^			
Yes	24 (80)	28 (93.3)	*X* ^2^ = 2.30, 1, *p* = 0.129
No	6 (20)	2 (6.7)	

*Number of grafts*			
2	1 (3.3)	1 (3.3)	*X* ^2^ = 0.07, 2, *p* = 0.962
3	10 (33.3)	9 (30)	
≥4	19 (63.4)	20 (66.7)	
*Number of chest tubes*			
2	24 (80)	27 (90)	*X* ^2^ = 1.17, 1, *p* = 0.278
3	6 (20)	3 (10)	

	(Mean ± SD)	(Mean ± SD)	*p* value ^*∗∗*^

Age (years)	60.80 ± 9.4	62.37 ± 10.1	*t*(58) = −0.61, *p* = 0.539
BMI (kg/m2)	26.73 ± 4.4	26.03 ± 4.1	*t*(58) = 0.63, *p* = 0.526
Smoking (butts/day)	4.73 ± 10.9	9.83 ± 15.3	*t*(52.41) = −1.48, *p* = 0.204
On-pump duration (minute)	109.70 ± 25.3	111.90 ± 28	*t*(58) = −0.32, *p* = 0.751
Ejection fraction (%)	46.50 ± 11.2	50.83 ± 5.5	*t*(42.51) = −1.89, *p* = 0.065
Medical ventilation time (hour)	9.63 ± 4.2	10.93 ± 4.3	*t*(58) = −1.16, *p* = 0.249
Duration of preoperative hospitalization (day)	4.5 ± 2	5.53 ± 3.2	*t*(49.2) = −1.46, *p* = 0.150
Duration of postoperative hospitalization (day)	5.67 ± 1.4	6.03 ± 1.3	*t*(58) = −1.01, *p* = 0.315

RB: rhythmic breathing; BMI: body mass index;  ^*∗*^Chi-square test;  ^*∗∗*^independent *t*-test. ^*a*^Including: hypertension, hyperlipidemia.

**Table 2:** The comparisons of mean scores of the pain severity in the RB and control groups.

Day	Hours	Measurement times	Groups	Mean ± SD	Min	Max	value ^*∗*^
Fist day	9 a.m.	Before intervention	RB	2.37 ± 0.99	1	5	*t*(52.6) = 0.213, *p* = 0.832
			Control	2.30 ± 1.39	1	6	
		After intervention	RB	1.07 ± 0.64	0	3	*t*(58) = −2.90, *p* = 0.005
			Control	1.50 ± 0.5	1	2	
	9 p.m.	Before intervention	RB	2.10 ± 0.84	1	4	*t*(58) = 0.91, *p* = 0.363
			Control	1.90 ± 0.84	1	4	
		After intervention	RB	0.87 ± 0.50	0	2	*t*(57.2) = -4.78, *p* < 0.001
			Control	1.53 ± 0.57	1	3	

Second day	9 a.m.	Before intervention	RB	1.90 ± 0.54	1	3	*t*(40) = −040, *p* = 0.687
			Control	2 ± 1.23	1	5	
		After intervention	RB	0.83 ± 0.46	0	2	*t*(53.3) = −5.17, *p* < 0.001
			Control	1.57 ± 0.62	1	3	
	9 p.m.	Before intervention	RB	1.97 ± 0.49	1	3	*t*(54.3) = 1.58, *p* = 0.119
			Control	1.73 ± 0.64	1	3	
		After intervention	RB	0.97 ± 0.49	0	2	*t*(55.5) = −4.91, *p* < 0.001
			Control	1.67 ± 0.60	1	3	

Third day	9 a.m.	Before intervention	RB	1.73 ± 0.58	1	3	*t*(58) = 1.78, *p* = 0.079
			Control	1.47 ± 0.57	1	3	
		After intervention	RB	0.70 ± 0.53	0	2	*t*(58) = −5.46, *p* < 0.001
			Control	1.43 ± 0.50	1	2	
	9 p.m.	Before intervention	RB	1.77 ± 0.62	1	3	*t*(58) = 2.27, *p* = 0.027
			Control	1.43 ± 0.50	1	2	
		After intervention	RB	0.70 ± 0.53	0	2	*t*(58) = −5.46, *p* < 0.001
			Control	1.43 ± 0.50	1	2	

RB : rhythmic breathing; SD: standard deviation; Min : minimum; Max : maximum;  ^*∗*^independent *t*-test.

**Table 3:** The comparison of the patients in the RB and control groups in terms of pain severity.

Day	Hours	Measurement times	Groups	No pain	Mild pain	Moderate pain	*p* value ^*∗*^
Fist day	9 a.m.	Before intervention	RB	0	25	5	*X* ^2^ = 0.109, 1, *p* = 0.741
			Control	0	24	6	
		After intervention	RB	4	26	0	*X* ^2^ = 4.21, 1, *p* = 0.040
			Control	0	30	0	
	9 p.m.	Before intervention	RB	0	27	3	*X* ^2^ = 1.05, 1, *p* = 0.305
			Control	0	29	1	
		After intervention	RB	6	24	0	*X* ^2^ = 6.55, 1, *p* = 0.010
			Control	0	30	0	

Second day	9 a.m.	Before intervention	RB	0	30	0	*X* ^2^ = 5.36, 1, *p* = 0.052
			Control	0	25	5	
		After intervention	RB	6	24	0	*X* ^2^ = 6.55, 1, *p* = 0.010
			Control	0	30	0	
	9 p.m.	Before intervention	RB	0	30	0	—
			Control	0	30	0	
		After intervention	RB	4	26	0	*X* ^2^ = 4.21, 1, *p* = 0.040
			Control	0	30	0	

Third day	9 a.m.	Before intervention	RB	0	30	0	—
			Control	0	30	0	
		After intervention	RB	10	20	0	*X* ^2^ = 11.80, 1, *p* = 0.001
			Control	0	30	0	
	9 p.m.	Before intervention	RB	0	30	0	—
			Control	0	30	0	
		After intervention	RB	10	20	0	*X* ^2^ = 11.80, 1, *p* = 0.001
			Control	0	30	0	

RB: rhythmic breathing;  ^*∗*^Chi-square (linear-by-linear association).

**Table 4:** The comparison of mean differences scores of the pain severity before and after intervention in the RB and control groups.

Day	Measurement times	Groups	Mean ± SD	Min	Max	*p* value ^*∗*^
Fist day	9 a.m.	RB	1.3 ± 0.59	1	3	*t*(45.5) = 2.24, *p* = 0.028
		Control	0.8 ± 1.06	0	4	
	9 p.m.	RB	1.23 ± 0.56	1	3	*t*(58) = 5.67, *p* < 0.001
		Control	0.36 ± 0.61	0	2	

Second day	9 a.m.	RB	1.06 ± 0.25	1	2	*t*(32.9) = 3.45, *p* = 0.002
		Control	0.43 ± 0.97	0	3	
	9 p.m.	RB	1/0 ± 0.0	1	1	*t*(29) = 20.15, *p* < 0.001
		Control	0.06 ± 0.25	0	1	

Third day	9 a.m.	RB	1.03 ± 0.18	1	2	*t*(58) = 21.2, *p* < 0.001
		Control	0.03 ± 0.18	0	1	
	9 p.m.	RB	1.06 ± 0.25	1	2	*t*(29) = 23, *p* < 0.001
		Control	0.0 ± 0.0	0	0	

RB : rhythmic breathing; SD: standard deviation; Min : minimum; Max : maximum;  ^*∗*^independent *t*-test.

**Table 5:** Results of repeated-measures ANOVA in terms of pain severity in the RB and control groups.

Source of change	Variables	Sum of squares	df ^*∗*^	Mean squares	F	*p* value
Within-subjects	Time^a^	13.10	1	13.10	10.56	0.002
	Time × group	3.66	1	3.66	2.95	0.09
	Error	71.90	58	1.24		

Between-subject	Constant	176.4	1	176.4	387.05	<0.001
	Group	62.50	1	62.50	137.13	<0.001 ^*∗∗*^
	Error	26.43	58	0.456		

^*∗*^Lower bound;  ^*∗∗*^adjusted for time, and interaction of time and group. Time^*a*^: day 1 (*T*1, *T*2); day 2 (*T*1, *T*2); and day 3 (*T*1, *T*2). T1 indicates the difference in pain severity between before and after the intervention at 9 a.m., and *T*2 indicates the difference in pain severity between before and after the intervention at 9 p.m..

## Data Availability

The datasets used and/or analyzed during the current study are available from the corresponding author on reasonable request.
